# Characterisation of a university student sample with a lifetime history of non-suicidal self-injury: mixed-methods analysis of stress factors, coping mechanisms and reasons for self-injury

**DOI:** 10.1192/bjo.2025.10893

**Published:** 2025-11-14

**Authors:** Elena von Perponcher, Irina Jarvers, Angelika Ecker, Elisa Heidingsfelder, Stephanie Kandsperger, Romuald Brunner, Daniel Schleicher

**Affiliations:** Institute for Experimental Psychology, University of Regensburg, Regensburg, Germany; Department of Child and Adolescent Psychiatry and Psychotherapy, University of Regensburg, Regensburg, Germany

**Keywords:** Non-suicidal self-injury, trauma and stressor-related disorders, depressive disorders, qualitative research, coping

## Abstract

**Background:**

Non-suicidal self-injury (NSSI) displays an alarmingly high prevalence rate among university students, placing them at high risk for adverse long-term outcomes, including suicide.

**Aims:**

This study aimed to achieve a better understanding of factors contributing to NSSI in university student populations by examining reasons for NSSI and histories of stressful events and coping strategies.

**Method:**

A total of 185 university students with a lifetime history of NSSI were assessed for depressive symptoms and NSSI characteristics. They completed three questionnaires on NSSI reasons, stressful events and coping strategies during childhood and adolescence. Each questionnaire included an ‘others’ option combined with an open-ended response box. After descriptive analysis of the closed questions, these open-ended responses were qualitatively categorised and analysed as predictors of depression severity and NSSI continuation from adolescence into adulthood.

**Results:**

Qualitative analysis identified eight, five and ten categories from the open-ended responses for NSSI reasons, stressful events and coping strategies, respectively, with substantial to almost perfect interrater reliability. Two qualitative reason categories, one stressful event category and two coping strategy categories significantly predicted depression severity (*β* = 0.21–0.23). Participants reporting events in the stressful events category ‘Traumatisation and experiences of violence’ were three times more likely to continue NSSI into adulthood (*f* ^2^ = 0.07).

**Conclusions:**

This study demonstrates the value of mixed-methods approaches. Stable qualitative categories highlight the need to capture individual variations in NSSI-related factors. It emphasises trauma-related stressors due to their influence on depression severity and persistence of NSSI into adulthood.

Non-suicidal self-injury (NSSI) refers to the deliberate, direct destruction of one’s own body tissue (e.g. self-cutting, -hitting) without conscious suicidal intent.^1,2^ It is distinct from self-harm, a broader term including both suicidal behaviour and indirect damage to one’s own body (e.g. taking overdoses).^2^ NSSI occurs in clinical and non-clinical populations, with prevalence rates ranging from 7.5 to 46.5% in adolescence, and 4.0 to 23.0% in adulthood.^3^ However, these reported rates vary considerably depending on the measurement methods employed^4^ and appear to be moderated by cultural and demographic factors such as race, socioeconomic status and gender.^5^ The purpose of NSSI usually is to resolve negative emotions, a negative cognitive state or interpersonal difficulties.^1^ NSSI is associated with life stress^6^ and diverse mental illnesses like borderline personality disorder and depression.^3^ Even though, by definition, these injuries do not carry suicidal intent, NSSI exhibits a reliable relationship with attempted suicide.^7^ Many individuals engaging in NSSI in adolescence cease NSSI in adulthood, and there is a lower likelihood of continued NSSI with increasing age.^8,9^ NSSI is more likely to be carried from adolescence into adulthood if it served intrapersonal functions (e.g. affect regulation or self-punishment^10^) in adolescence,^8^ and less likely to be carried on with higher perceived emotion regulatory capability.^11^ Treatment approaches like dialectical or cognitive behaviour therapy have been shown to effectively reduce NSSI, suicidal ideation and suicide attempt rates.^12–14^ Among non-clinical populations, university students display one of the highest NSSI prevalence rates, at an alarming 38.9%.^3^ University students not only face a range of intense emotional, social and academic demands that can contribute to psychological distress,^15^ they also often exhibit low treatment-seeking behaviours,^16^ putting them at an even greater risk for suicide and other negative mental health outcomes. Given the gravity of this issue, understanding the factors contributing to NSSI in this population is essential and may offer important implications for interventions, e.g. within campus mental health services. Well-established questionnaires can be employed to evaluate and characterise NSSI quantitatively. Some incorporate an ‘others’ category (e.g.^17,18^), acknowledging that the predefined response options may not fully encompass all NSSI-related factors relevant to one individual. However, the information driving these ‘others’ responses usually is not investigated further, potentially resulting in the loss of valuable information. Qualitative methodology approaches have investigated the topic of self-injury through semi-structured interviews (e.g.^19,20^) or open-ended questions (e.g.^18,21,22^). However, integrating both quantitative and qualitative methods, especially within questionnaires, enables a more comprehensive and contextualised understanding of NSSI-associated factors. To the best of our knowledge, no prior study has employed a mixed-methods approach to investigate multiple NSSI-related factors in university students. Therefore, this study utilised questionnaires to examine NSSI characteristics, reasons, stress factors and coping mechanisms in a university sample with a lifetime history of NSSI. These questionnaires included an ‘others’ response option with the option to provide further information in free-text format. We aimed to investigate NSSI-related factors through a combined quantitative and qualitative approach and sought to identify predictors-for-depression score levels as well as risk factors for the continuation of NSSI from childhood into adolescence within categories that emerged qualitatively from the ‘others’ response option.

## Method

### Study design and participants

This study analysed data from a large online survey of university students with a lifetime history of NSSI. Participants were recruited through notices, flyers, mailing lists and online advertisements. The survey was conducted on the PsyToolkit online platform^23,24^ from April to September 2021 and included students aged 18 to 25 who had a sufficient understanding of the German language and a history of NSSI. Participants were excluded only if they failed to meet inclusion criteria. Upon completion, participants could enter a draw for 25 gift vouchers worth 15€ each.

A total of *N* = 240 students participated in the survey; however, *n* = 51 were excluded as they did not complete any of the relevant questionnaires. Additionally, *n* = 4 were excluded as they denied a history of NSSI, resulting in a final sample of *n* = 185 with a mean age of 21.88 years (s.d. = 1.94, range: 18–25).

Most of the final sample identified as female (*n* = 158), one student identified as diverse. Regarding NSSI history, 27.03% reported engaging in NSSI only during adolescence, 66.49% continued NSSI from adolescence (under 18 years) into adulthood (18 years and older) and 6.49% experienced NSSI solely in adulthood. At the time of the study, 20.54% were actively engaging in NSSI. A total of 98.92% had self-injured more than once in their life, 73.51% reported engaging in NSSI more than five times during their most severe year and 18.92% reported at least one previous suicide attempt. Regarding treatment history, 9.73% had required admission or medical treatment after NSSI at least once, and 36.22% had received psychiatric, psychotherapeutic or psychological treatment due to NSSI. Regarding study programmes, 55.14% were enrolled in a bachelor’s programme, 14.05% in a master’s programme and 30.81% were studying for a state exam. Regarding disciplines, 17.84% were studying to be teachers, 15.14% studied natural sciences, 9.73% social sciences, 9.19% psychology, 9.19% medicine, 6.49% economics, 5.95% law, 2.70% engineering and 20.54% other subjects. In addition, 3.24% chose to not disclose their field of study but to continue with the survey.

### Data collection procedure and measures

Students provided digital informed consent after being informed of the inclusion criteria and study objectives. Trigger warnings and contact information of counselling resources and study staff (research assistants, psychologists and psychiatrists) were displayed regularly throughout the online survey, including links to university counselling centres, emergency psychiatric services, 24/7 crisis helplines and online support platforms (chat, e-mail or in-person). Participation was anonymous and voluntary, and students could withdraw at any time without explanation by exiting the survey.

After providing informed consent, participants were asked to report demographic information (e.g. gender, age and study programme) and the key characteristics of NSSI, including its duration and frequency. In addition, information on the history of suicide attempts was collected. Participants then completed a series of questionnaires addressing NSSI-related factors.

Utilising parts of a German version of the Functional Assessment of Self-Mutilation (FASM,^25,26^), 22 different reasons for the engagement in NSSI at any time in the participants’ lives were queried. The closed questions assessed the frequency of self-harm reasons (0 = ‘never’, 3 = ‘often’). From the 22 reasons, four reinforcement scales were calculated, based on a four-factor model of NSSI functions.^26^ These scales encompassed automatic-negative reinforcement (e.g. ‘to stop bad feelings’), automatic-positive reinforcement (e.g. ‘to feel relaxed’), social-negative reinforcement (e.g. ‘to avoid being with people’) and social-positive reinforcement (e.g. ‘to get control over a situation’).^27^ A 23rd ‘others’ response option, followed by the option to enter one or more reasons manually, allowed participants to provide additional reasons not covered by the structured questions.

The German version of the Brief Patient Health Questionnaire (PHQ-9)^28,29^ was used to screen for depressive symptoms, by analysing the sum score of nine exclusively closed questions assessing symptom frequency (from 0 = ‘not at all’ to 3 = ‘nearly every day’) over the past two weeks.

To investigate stressful life events in childhood and adolescence, and the coping strategies employed to manage these events, we self-constructed questionnaire items due to the lack of suitable German-language questionnaires. These items were based on the Survey of Stress and Coping in Childhood and Adolescence^30^ and the Children’s Inventory of Everyday Stressors (Inventario Infantil de Estresores Cotidianos).^31^ The questionnaire featured closed questions on five stressful event and coping mechanism types, each with an ‘others’ option paired with a free-text box for additional responses. Participants rated the intensity of stressful events and the helpfulness of coping mechanisms from 0 (‘not at all’) to 10 (‘very’). Table 1 provides an overview of the questions.

**Table 1:** Questions and response options of the self-constructed stressful events and coping mechanisms questionnaires

Stressful events	
Category	Question: In your childhood and adolescence, were there any stressful events …
Family and home	… in the family and home (e.g. separation/divorce of parents, loss of caregivers, parental absence, serious illness of a family member, etc.)?
Friends and peers	… involving friends and peers (e.g. bullying, exclusion, manipulation, threats or similar)?
School and education	… in the area of school and education (e.g. lack of equal rights, excessive demands, bullying, failures or similar)?
Physical area	… in the physical area (e.g. nutritional problems, over/underweight, worries about appearance/complexes, excessive tiredness or similar)?
Health	… in the area of health (e.g. chronic illness, disability, pain, accident, operation or similar)?
Others	… in any other areas?
Coping mechanisms	
Category	Question: How did you cope with these stressors?
Support	I sought support from others (e.g. talked to others about it, sought/accepted help from others, allowed myself to be comforted or similar)
Problem tackling	I actively tackled the existing problem (e.g. looked for/implemented solutions, changed the situation to improve it in the future or similar)
Problem avoidance	I avoided the existing problem (e.g. I distracted myself, played it down, did nothing or similar)
Recovery	I recovered (e.g. taken a break, gathered strength, relaxed, treated myself to something or similar)
Letting it out	I let out my bad mood (e.g. I was angry and upset, swore, slammed doors, got upset or similar)
Others	I dealt with it in other ways

All questions could be answered with ‘yes’ or ‘no’.

### Mixed-methods approach for data analysis

After extracting all qualitative data from the three questionnaires (ranging from 44 to 66 participants’ responses per questionnaire), one response was removed from the FASM data as that participant had listed ‘suicide’ as an NSSI reason, which falls outside NSSI criteria. Subsequently, we used an inductive coding strategy^32–35^ to analyse the open-ended ‘others’ response options of all three questionnaires, independently of the closed-ended questions. The inductive coding was performed by three raters (R1, R2 and R3), all native German speakers, who all held at least a master’s degree in psychology. Two of them (R2 and R3) were in advanced psychotherapist training. Each of the three response arrays was independently rated by two of the three raters (FASM: R1, R3; stress: R1, R2; coping: R1, R2). If participant statements could be sorted in multiple categories (e.g. if a participant gave a comprehensive list of various NSSI reasons), they were split up into multiple statements per person to allow for clean categorisation. The two raters who scored the same questionnaire then discussed their individually extracted categories and statement-sorting to accomplish a joint data categorisation.^34^ A fourth rater (R4), a university professor and medical doctor with a psychiatry specialisation, and a fifth rater (R5), a medical doctor with a psychiatry specialisation, both highly experienced in clinical psychology and psychiatry research, then reviewed and approved the data categorisation across all questionnaires.^35^ Subsequently, the third rater who had not previously worked on the respective questionnaire (FASM: R2; stress: R3; coping: R3) was provided with the final statements and categories for that questionnaire and sorted the statements into the corresponding categories.^32^ This allowed us to compute interrater reliability metrics between the joint categorisation of the initial two raters and the categorisation of the third rater. Percentage agreements (the number of statements sorted correctly divided by the number of all statements) and Cohen’s kappa agreement coefficients *κ*^36^ were calculated for each questionnaire and each individual category.

The *κ* values were interpreted in accordance with established guidelines (*κ* < 0.00 = ‘poor’; 0.00 < *κ* < 0.20 = ‘slight’; 0.20 < *κ* < 0.40 = ‘fair’; 0.40 < *κ* < 0.60 = ‘moderate’; 0.60 < *κ* < 0.80 = ‘substantial’; *κ* > 0.80 = ‘almost perfect’^37^). Where the third rater had difficulty clearly distinguishing between two categories (*κ* < 0.80), the initial two raters revisited the category descriptions and statement-sorting to refine them further.

After finalising the qualitative categories, potential thematic overlap with the closed-ended questionnaire items was examined. We chose not to re-code open-ended responses into corresponding closed-ended categories for two reasons: first, the frequency, intensity and helpfulness ratings for ‘other’ NSSI reasons, stressful events and coping mechanisms were assessed only once for the general ‘others’ item in each questionnaire. For participants who listed multiple responses in the free-text section, these general ratings would likely not have accurately reflected the frequency, intensity or helpfulness of each individual statement. Second, we aimed at identifying closed-ended items where participants felt their NSSI-related factors were inadequately represented, e.g. due to unclear concept definition in the items. Where substantial overlap occurred, we further analysed the number of participants who did not select the relevant closed-ended category but mentioned something in the ‘others’ section that was later categorised into the similar qualitative category.

### Statistical analyses

Based on both closed and open-ended questions, statistical analyses with *α* = 0.05 were conducted using IBM SPSS Statistics (version 29.0 for Windows, IBM Corp., Armonk, NY, USA). Each analysis was conducted only for the subsample that had completed the relevant questionnaire(s). Descriptive analyses identified relevant patterns in reasons for NSSI, stressors and coping strategies. Friedman tests examined differences in perceived stressfulness across the qualitatively derived stressful event categories. To assess links between qualitative categories and depression severity (PHQ-9), exploratory Mann–Whitney U tests were conducted to compare PHQ-9 scores for participants reporting something in a given category versus those who did not (either by not selecting the ‘others’ option initially, or by selecting it without subsequently specifying something later sorted into that category). Effect sizes were interpreted in accordance with established guidelines (0.10 < *r* < 0.30 = ‘small’; 0.30 < *r* < 0.50 = ‘medium’ ; *r* ≥ 0.50 = ‘large’^38^). Significant differences prompted linear regression with the significant categories as predictors and the PHQ-9 scores as the dependent variable, to further analyse the impact of these categories on depression scores. To assess links between qualitative categories and NSSI continuation into adulthood, 12 participants reporting engaging in NSSI only during adulthood were not included in the analysis, leaving a sample of *n* = 173. Exploratory *χ*^2^ tests (applying Yate’s continuity correction) assessed whether participants who indicated something in one category were more likely to belong to the continuation (cNSSI) or discontinuation (dNSSI) group. For further analysis of statistically significant categories, logistic regression models were employed with these categories as predictors and the cNSSI/dNSSI groups as the dependent variable.

## Results

### FASM results

**Fig. 1:**
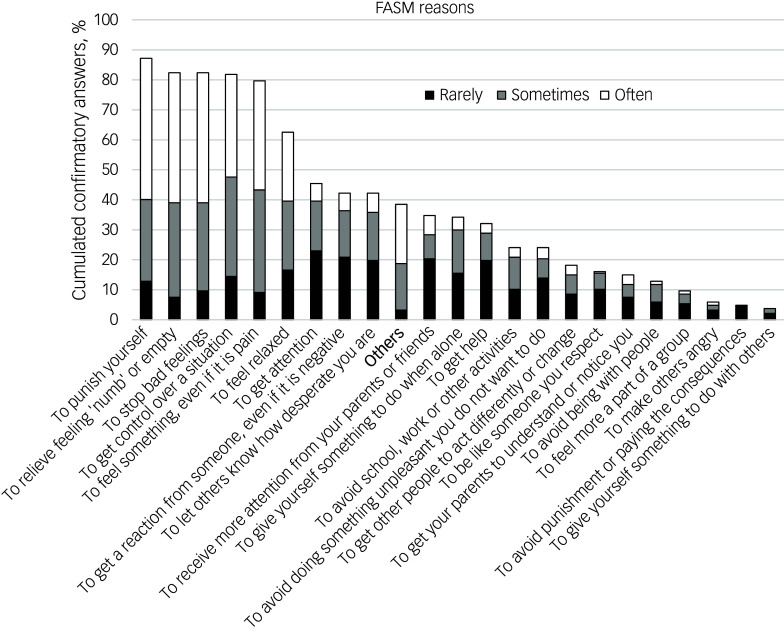
Cumulative percentages of participants who confirmed having engaged in non-suicidal self-injury due to one given reason. FASM, Functional Assessment of Self-Mutilation. Sorted from the most to the least frequently reported reason, broken down by the confirmative response options ‘rarely’, ‘sometimes’ and ‘often’. The remaining percentages up to 100% represent the frequency of the response option ‘never’.

Overall, 185 participants completed the FASM, with scores across the 22 given reasons and the ‘others’ option shown in Fig. 1. Reinforcement scale calculations indicated automatic negative (mean (*M*) = 1.99, s.d. = 0.89) and automatic positive reinforcement (*M* = 1.74, s.d. = 0.78) as the most common functions, followed by social positive (*M* = 0.51, s.d. = 0.44) and social negative reinforcement (*M* = 0.25, s.d. = 0.42). The ‘others’ option was selected by 72 participants (38.50% of the FASM completion sample), with 90.05% subsequently entering one or more NSSI reasons manually. Qualitative analysis of these reasons identified eight categories with 81% agreement and *κ* = 0.79. Table 2 presents the final categories for the FASM ‘others’ option, with percentage agreements and *κ* for each individual category.

**Table 2:** Categories emerged from the qualitative analysis of the open-ended response option of the Functional Assessment of Self-Mutilation (FASM)

Category name	Example statements	Participants, *n* (percentage of sample^a^)	Agreement, %^b^	Cohen’s kappa^b^
Occurrence of or dealing with something negative	‘grief’; ‘to deal with emotional overload’	27 (14.59)	83	0.80
Termination or reduction of something negative	‘to stop flashbacks’; ‘to relieve pressure’	16 (8.65)	88	0.88
Bringing about or maintaining something subjectively positive	‘to be able to focus on studying’; ‘to have access to my own body’	10 (5.41)	96	0.96
Distraction from, avoidance of or escape from something negative	‘to distract myself from loneliness’; ‘to escape feelings of desperation’	6 (3.24)	99	0.99
Automatism, habit or impulsivity	‘it became a hobby’; ‘habit’; ‘unconscious impulsive action’	6 (3.24)	99	0.99
Self-deprecation or self-punishment	‘to hurt myself’; ‘to realise that I am a bad person’	5 (2.70)	97	0.97
Aesthetics	‘I found the wounds beautiful’	3 (1.62)	100	1.00
Adaptation to peer group	’suggested handling of problems by former peer group’	1 (0.54)	99	0.99

a. ’Sample’ is all participants that completed the FASM questionnaire.

b. Calculated between the joint categorisation of the initial two and the categorisation of the respective third rater, for each individual category.

Review of the final qualitative categories revealed overlap with the closed-ended FASM items, particularly between the qualitative category ‘Termination or reduction of something negative’ and the item ‘to stop bad feelings’, and between ‘Self-deprecation or self-punishment’ and ‘to punish yourself’. The numbers of participants that indicated something in the qualitative category, but had not selected the relevant closed-ended category before, were two and zero for the first and second overlap pair, respectively.

### Stressful events results

**Fig. 2:**
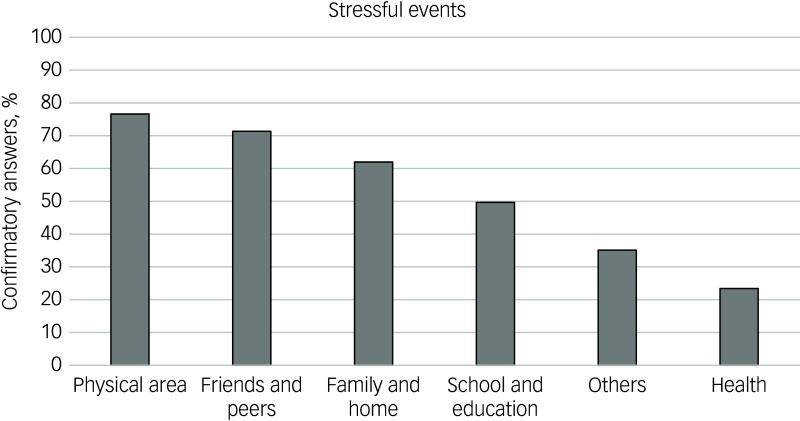
Cumulated percentages of participants confirming one given category of stressful events. Sorted from the most to the least frequently reported stressful event item.

A total of 170 participants completed the stressful events questionnaire, with frequencies across the five given questionnaire categories and the ‘others’ option shown in Fig. 2. The median (Q1/Q3) perceived stressfulness scores were 6.00 (4.00/9.00) for physical area, 7.00 (6.00/8.00) for friends and peers, 8.00 (5.00/10.00) for family and home, 7.00 (4.00/8.00) for school and education, 9.00 (7.00/10.00) for others and 6.00 (3.00/9.00) for health. No significant differences in stressfulness were found across the six categories (*χ*^2^(5) = 5.74; *p* = 0.332). The ‘others’ option was selected by 60 participants (35.29% of the subsample that completed the questionnaire), with 93.33% subsequently entering one or more stressful events manually. Qualitative analysis of these events identified five categories with 86% agreement and *κ* = 0.80. Table 3 presents the final categories for the stressful events ‘others’ option, with percentage agreements and *κ* for each individual category.

**Table 3:** Categories emerged from the qualitative analysis of the open-ended response option of the self-constructed stressful event questionnaire

Category name	Example statements	Participants, *n* (percentage of sample^a^)	Agreement, %^b^	Cohen’s kappa^b,c^
Traumatisation or experiences of violence	‘aggressive and abusive brother’; ‘I got hit by my father’; ‘rape’	28 (16.47)	96	0.92
Dealing with own distressing emotions, negative basic assumptions or behavioural problems	‘feeling of being different; ‘loneliness’; ‘drug abuse’; ‘identity crisis’	14 (8.24)	93	0.79
Interactions, changes or existential difficulties in the social and family system	‘upbringing in a sect’; ‘sibling rivalry’; ‘my father lost his company’	13 (7.65)	86	0.63
Illness or loss of close persons	‘a close friend’s cancer’; ‘death of two close contact persons’	8 (4.71)	100	1.00
Others	‘political extremism’; ‘my premature birth’	2 (1.18)	96	–

a. ’Sample’ is all participants that completed the stressful events questionnaire.

b. Calculated between the joint categorisation of the initial two and the categorisation of the respective third rater, for each individual category.

c. No statistically meaningful Cohen’s kappa coefficient could be calculated for the ‘Others’ category, as none of the statements was scored as ‘Others’ by the third rater.

Thematic overlap with closed-ended items occurred between ‘Interactions, changes or existential difficulties in the social and family system’ and ‘Family and home’. Of the 13 participants, 5 indicating a stressful event later sorted into the qualitative category had not selected the closed-ended item ‘Family and home’ before.

### Coping strategy results

**Fig. 3:**
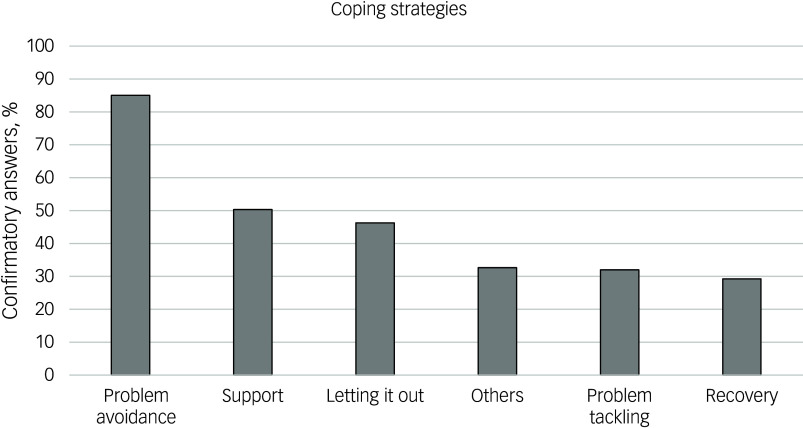
Cumulated percentages of participants confirming one given category of coping strategies. Sorted from the most to the least frequently reported coping strategy items.

Overall, 147 participants completed the coping strategy questionnaire, with frequencies across the 5 given questionnaire categories and the ‘others’ option shown in Fig. 3. The median (Q1/Q3) perceived helpfulness scores were 4.00 (2.00/6.00) for problem avoidance, 7.00 (4.00/9.00) for support, 5.00 (2.50/7.00) for letting it out, 6.00 (2.50/8.00) for others, 6.00 (4.00/8.00) for problem tackling and 6.00 (4.00/8.00) for recovery. Friedman test calculations were not possible due to an insufficient number of participants confirming all six closed-question categories. The ‘others’ option was selected by 48 participants (32.65% of the subsample that completed the questionnaire), with 91.67% subsequently entering one or more coping strategies manually. Qualitative analysis of these strategies identified 10 categories with 79% agreement and *κ* = 0.97. Table 4 presents the final categories for the coping strategies ‘others’ option, with percentage agreements and *κ* for each individual category.

**Table 4:** Categories emerged from the qualitative analysis of the open-ended response option of the self-constructed coping strategies questionnaire

Category name	Example statements	Participants, *n* (percentage of sample^a^)	Agreement, %^b^	Cohen’s kappa^b^
Auto-aggression	’self-injury’; ‘self-hatred’; ‘suicide attempt’	18 (12.24)	100	1.00
Substance use	‘alcohol’; ‘cigarettes’; ‘drugs’	9 (6.12)	100	1.00
Withdrawal, isolation or independent coping	‘isolation’; ‘I tried to deal with the problems myself’; ‘I built up my own world’	7 (4.76)	97	0.99
Coping through positively experienced or structuring activities	’sports’; ‘work’; ‘reading’	7 (4.76)	97	0.97
Suppression, denial or avoidance	‘I avoided stressful situations’; ‘ignorance’; ‘suppressed emotions’	6 (4.08)	100	1.00
Seeking professional or social support	‘therapy’; ‘talked to an adult’	5 (3.40)	100	1.00
Change in eating behaviour	‘control through food (anorexia)’; ‘excessive food consumption’	5 (3.40)	100	1.00
Externalisation	‘I picked up fights’; ‘I was moody’	4 (2.72)	100	1.00
Endurance and resignation	‘I suffered through it’; ‘resignation’	3 (2.04)	100	1.00
Overcompensation	‘I tried to do everything right’; ‘humour’	2 (1.36)	99	0.99

a. ’Sample’ is all participants that completed the coping strategies questionnaire.

b. Calculated between the joint categorisation of the initial two and the categorisation of the respective third rater, for each individual category.

Thematic overlap could be identified for ‘Suppression, denial or avoidance’ with ‘Problem avoidance’, ‘Seeking professional or social support’ with ‘Support’, and ‘Externalisation’ with ‘Letting it out’. In the first pair, all participants who indicated responses in the qualitative category had already selected the corresponding quantitative category. In the other two pairs, only one participant each fit that pattern.

### Predictors of depression

#### FASM

With *n* = 185 participants completing the FASM, Mann–Whitney U tests revealed significantly higher PHQ-9 scores for participants endorsing one or more reasons in ‘Termination or reduction of something negative’ (*U* = 1722.00; *z* = 2.62; *p* = 0.009; *r* = 0.20) and/or ‘Self-deprecation or self-punishment’ (*U* = 647.50; *z* = 2.17; *p* = 0.030; *r* = 0.17). The multiple regression model using both categories as predictors was significant (*F*(2,167) = 6.20; *p* = 0.003), explaining 5.8% of PHQ-9 score variance. Both ‘Termination or reduction of something negative’ (*β* = 0.21; *p* = 0.006) and ‘Self-deprecation or self-punishment’ (*β* = 0.17; *p* = 0.023) were significant predictors of the PHQ-9 score (see Table 5).

**Table 5:** Overview of qualitative categories predicting the PHQ-9 score in linear regressions

Questionnaire	Predictors	*B*	s.e.	95% CI	*β*	*t*	*p*
Functional Assessment of Self-Mutliation	‘Termination or reduction of something negative’	4.71	1.69	1.38–9.04	0.21	2.79	0.006
	’Self-deprecation or self-punishment’	6.70	2.92	0.95–12.45	0.17	2.30	0.023
Stressful events	‘Traumatisation or experiences of violence’	3.86	1.34	1.22–6.50	0.22	2.89	0.004
Coping strategies	‘Auto-aggression’	4.55	1.61	1.38–7.72	0.23	2.83	0.005
	‘Withdrawal, isolation or independent coping’	6.42	2.47	1.54–11.31	0.21	2.60	0.010

#### Stressful events

Among *n* = 170 participants completing the stressful events questionnaire, Mann–Whitney U tests revealed significantly higher PHQ-9 scores only for participants indicating one or more events in ‘Traumatisation or experiences of violence’ (*U* = 2647.00; *z* = 2.77; *p* = 0.006; *r* = 0.21). The linear regression model with this category as predictor was significant (*F*(1,169) = 8.33; *p* = 0.004; *β* = 0.22), explaining 4.7% of PHQ-9 score variance (see Table 5).

#### Coping strategies

With *n* = 147 participants completing the coping strategies questionnaire, Mann–Whitney U tests revealed significantly higher PHQ-9 scores for people who had indicated one or more strategies in ‘Auto-aggression’ (*U* = 1621.50; *z* = 2.72; *p* = 0.006; *r* = 0.22) and/or ‘Withdrawal, isolation or independent coping’ (*U* = 767.00; *z* = 2.52; *p* = 0.012; *r* = 0.21). The multiple regression model using both categories as predictors was significant (*F*(2,144) = 7.50; *p* < 0.001), explaining 8.2% of variance in PHQ-9 score variance. Both ‘Auto-aggression’ (*β* = 0.23; *p* = 0.005) and ‘Withdrawal, isolation or independent coping’ (*β* = 0.21; *p* = 0.010) were significant predictors of the PHQ-9 score (see Table 5).

For linear regressions, the minimum detectable effect sizes were *f* ^2^ = 0.09 for FASM (8 predictors), *f* ^2^ = 0.08 for stressful events (5 predictors), and *f* ^2^ = 0.12 for coping strategies (10 predictors). However, due to prior correlation analyses, only a maximum of two predictors were included in each regression model, allowing for the detection of smaller effects: *f* ^2^ = 0.06 for FASM and *f* ^2^ = 0.07 for coping strategies.

### Predictors of NSSI continuation

Yates-corrected *χ*^2^ tests showed no significant impact of any FASM or coping strategy categories on the continuation of NSSI into adulthood, as opposed to the stressful events category ‘Traumatisation or experiences of violence’ (*χ*^2^(158) = 6.12; *p* = 0.025). A binomial logistic regression model with this category as predictor and the cNSSI/dNSSI groups as the dependent variable was significant (*χ*^2^(1) = 6.23; *p* = 0.013), classifying 69.6% of participants correctly. It explained 6.2% of group assignment variance (Nagelkerke *R*^2^), with a small effect size (*f* ^2^ = 0.07^39^). Participants who had indicated one or more stressful events in the ‘Traumatisation or experiences of violence’ category, were 300.10% more likely to belong to the cNSSI group.

To detect a medium effect size (*w* = 0.30) with a *χ*^2^ test at 80% power and *α* = 0.05, a sample size of approximately 87 participants was required. With a sample size of 147, the 2 × 2 *χ*^2^ test (*α* = 0.05, power 0.80) was able to detect an effect size of approximately *w* = 0.23, which is considered small-to-medium. Given our sample size of 170 participants and an event rate of 16.48%, the logistic regression analysis was sufficiently powered (80%) to detect an odds ratio of approximately 2.08, which is considered medium-to-large.

## Discussion

This study examined NSSI reasons as well as stressful events and coping strategies during childhood and adolescence in university students with a history of NSSI. Using a mixed-methods approach with three questionnaires, the closed-ended questions were analysed quantitatively, while the free-text responses from the ‘others’ options were categorised qualitatively. These categories were further analysed to explore their relationship with depression scores and the persistence of NSSI from adolescence into adulthood.

The ‘others’ option ranked 10th out of 23 categories for the FASM questionnaire, and 5th and 4th out of 6 for the stressful events and coping strategies questionnaires, respectively. Across all questionnaires, over 90% of participants who selected the ‘others’ option provided additional information in the subsequent open-response section. These findings indicate a significant need for participants to express information beyond the preceding closed-ended questions, likely because they felt their individual NSSI-related factors were not adequately represented by the provided closed-ended response options. This highlights the importance of mixed-methods approaches to allow for more individualised and detailed investigations of NSSI.

### Reasons for NSSI (FASM)

In the present study, five of the six most commonly reported closed-ended NSSI reasons fell under positive or negative automatic reinforcement functions, aligning with prior research highlighting the strong automatic, intrapersonal functions of NSSI in institutionalised adolescent samples.^26,40^ For comparisons with other studies, the ‘others’ option of this study was excluded from the ranking, as the studies used for comparison did not include a comparable response option. The endorsement pattern of the remaining 22 closed-ended NSSI reasons resembled the pattern recently identified in Swedish high school students with a history of NSSI,^41^ showing rank differences of no more than 4 positions. However, it differed considerably from FASM reason findings reported for Indian college student^42^ and USA adolescent^27^ samples exhibiting minor to severe NSSI behaviours. These differences were particularly pronounced in the categories ‘to avoid feeling “numb” or empty’ (present study: rank 2; other studies (India and USA, respectively): ranks 15 and 11), ‘to let others know how desperate you are’ (present study: rank 9; other studies: ranks 14 and 20), ‘to get help’ (present study: rank 12; other studies: ranks 17 and 22) and ‘to avoid school, work or other activities’ (present study: rank 13; other studies: ranks 6 and 21). These discrepancies may at least partly stem from differences in the investigated NSSI timeframe (Indian and US studies: over the past year; Swedish study: over the past year or at any time previously; present study: at any time). Additionally, cultural differences in norms and attitudes towards mental health, emotions, coping and the social acceptability of specific NSSI reasons may play a role.

More than half of the participants who indicated ‘other’ reasons for NSSI in the present study reported that these reasons occurred frequently. This contrasts with similarly sized categories from the closed-ended FASM questions, where the primary reported frequency was ‘rarely’. This difference in frequency relations further underscores the importance of considering the ‘others’ category answers.

#### Thematic overlap

Thematic overlap was detected between the qualitative category ‘Termination or reduction of something negative’ and the closed-ended item ‘to stop bad feelings’, as well as between ‘Self-deprecation or self-punishment’ and ‘to punish yourself’. The low number of participants in these overlaps that had not selected the closed-ended category before indicating something in the similar qualitative category suggests that certain recurring themes in the qualitative analysis likely reflect inadequate or imprecise representation of NSSI reasons in the closed-ended questions of the FASM. For instance, reasons such as ‘to stop panic attacks’ or ‘to reduce tension’ were not viewed as fitting within the closed-ended category ‘to stop bad feelings’, possibly because ‘panic attacks’ were not perceived as a ‘feeling’, and ‘reducing’ something was not seen as equivalent to ‘ending’ it.

#### No thematic overlap

The largest qualitative category, ‘Occurrence of or dealing with something negative’, exceeded the size of five given closed-ended FASM categories, and did not thematically correspond with any of the closed-ended categories. We interpreted this missing overlap to mean that the participants in this category felt like the presence of the feeling itself triggered NSSI behaviour as a way of ‘dealing with the feeling’, and that ‘dealing with it’ was perceived differently than ‘ending’ it, as addressed by one of the closed-ended FASM items or explicitly ‘reducing’ it. This qualitative category shows similarities to the category ‘managing distress/affect regulation’ that was identified as a recurrent reason for NSSI in a review of quantitative and qualitative studies.^43^ The results strengthen evidence that NSSI frequently serves as an emotion regulation strategy,^44,45^ and highlight the need for further investigation into its specific mechanisms. To address this, the use of adequate questionnaires that examine NSSI as an emotion regulation strategy is essential to minimise the loss of critical information. Furthermore, in light of prior qualitative findings identifying the acquisition of emotion regulation strategies as central to NSSI cessation,^18^ psychotherapy aimed at reducing NSSI behaviours should prioritise the development of functional emotion regulation strategies following an individualised assessment of NSSI functions. Building on these findings, implementing screening and prevention strategies in university settings could be valuable. University counselling services could offer targeted support, both in-person and digitally, specifically addressing NSSI. Measures might include risk factor assessments, dedicated support programmes, awareness campaigns and increasing the visibility of available resources to help students access timely assistance.

### Stressful events

The most frequently reported closed-ended stressful event items were in line with previous research linking NSSI to stressful events related to the respective items ‘Physical area’ (e.g. overweight in adolescent girls^46^), ‘Friends and peers’ (e.g. bullying^47^), ‘Family and home’ (e.g. family dynamic^48^) and ‘School and education’ (e.g. academic achievement satisfaction^49^).

Thematic overlap was identified between ‘Interactions, changes or existential difficulties in the social and family system’ and ‘Family and home’. Notably, *κ* for the qualitative category was the lowest of all scores (0.63; substantial agreement). Review of the qualitative stressful events indicated in the qualitative category by the participants that had not initially chosen the closed-ended category ‘Family and home’ revealed that all indicated events (e.g. ‘sibling rivalry’) were closely related to the corresponding closed-ended category. A more precise definition of ‘Family and home’ in the questionnaire may have reduced this discrepancy.

No thematic overlap was found between the largest qualitative category, ‘Traumatisation or experiences of violence’, and any closed-ended categories, reflecting the well-established relationship between trauma and self-injury.^50^ Notably, all free-text entries exceeding the single word ‘trauma’ described sexual or emotional abuse (e.g. ‘rape’; ‘emotional abuse’), in line with results positioning these abuse forms during childhood as relevant impact factors for engagement in NSSI.^51^ The second largest qualitative category, ‘Dealing with own distressing emotions, negative basic assumptions or behavioural problems’, exclusively encompassed internal stressors, which were overlooked in the closed-ended questions on ‘Physical area’ and ‘Health’. The findings emphasise the importance of considering mental health alongside physical health as a potential source of stressors.

### Coping strategies

The primary closed-ended coping strategy, ‘Problem avoidance’, may not fully generalise to a gender-balanced student sample, as this study sample consisted predominantly of women, who were found to utilise avoidant coping styles more frequently than men.^52^ Nonetheless, these results are concerning, as maladaptive coping strategies like avoidance in college students are associated with negative mental and physical health outcomes.^53^

Thematic overlap was detected for ‘Suppression, denial or avoidance’ with ‘Problem avoidance’; ‘Seeking professional or social support’ with ‘Support’; and ‘Externalisation’ with ‘Letting it out’. The number of participants in these overlaps that had not selected the closed-ended category before indicating something in the similar qualitative category was low, suggesting that participants did not tend to use the open-ended response option to report coping mechanisms they felt were already covered by closed-ended categories.

No thematic overlap was identified for the remaining qualitative coping categories. The main qualitative category, ‘Auto-aggression’, mainly encompassing self-injurious behaviours, aligned with evidence of NSSI as a (coping) strategy for emotion regulation,^45^ and was unsurprising given the sample characteristics. Other categories (‘Endurance and resignation’, ‘Overcompensation’) mirrored schema therapy coping styles.^54^ Although all qualitative categories except ‘Auto-aggression’ were significantly smaller than the smallest closed-ended category, all exhibited very high *κ* values (all almost perfect agreement). This stability in categories like ‘Withdrawal, isolation or independent coping’, ‘Substance use’ and ‘Coping through positively experienced or structuring activities’, the latter two being equivalent or similar to coping mechanisms reported in a USA community college student sample,^55^ demonstrates recurring patterns of important adaptive and maladaptive coping mechanisms.

### Relationship of qualitative categories with depression scores and the persistence of NSSI from adolescence into adulthood

Two NSSI reasons, ‘Termination or reduction of something negative’ and ‘Self-deprecation or self-punishment’, were significant predictors of depression severity, supporting intrapersonal NSSI functions in depression.^56^ Furthermore, the largest qualitative stressful event category, ‘Traumatisation or experiences of violence’, significantly predicted depression scores, consistent with research positioning childhood emotional and sexual abuse as risk factors for adult depression.^57^ Moreover, two qualitative coping strategy categories, ‘Auto-aggression’ and ‘Withdrawal, isolation or independent coping’, also predicted depression severity. The first aligns with findings linking NSSI to later depression,^58^ while the latter is consistent with results of coping through withdrawal predicting adolescent symptoms across multiple years.^59^

The stressful events category ‘Traumatisation or experiences of violence’ was the only significant predictor of NSSI continuation from adolescence into adulthood. Participants who indicated something in this category were over three times more likely to belong to the group that continued NSSI. These findings align with the observation that decreased emotion regulation capacity, which is linked to traumatic experiences,^60^ is associated with the persistence of NSSI into adulthood.^8^

### Limitations and strengths of the study

This study utilised a university student sample from diverse academic backgrounds, with an age distribution reflecting the target population. Participant motivation and honesty were likely high due to the low financial incentive, anonymous online format and the stable response rates in the open-ended questions. The qualitative categorisation by multiple raters through careful individual ratings and group discussions demonstrated substantial to almost perfect interrater reliability (*κ* = 0.63 to 1.00) and was further validated by clinical experts, supporting the robustness of the categories. However, implicit biases may have influenced judgements, particularly for brief responses lacking context. While the study provides insights into the prevalence and correlates of NSSI, we did not assess the severity of NSSI behaviours in detail, which may at least partly differ in reason patterns.^27,42^ This limits our ability to differentiate between varying levels of NSSI severity and their associated factors. Future research should consider including standardised measures to capture the severity and functional aspects of NSSI. Moreover, the distinct sample characteristics – German-speaking, predominantly female university students – combined with a relatively small sample size, limit the generalisability of the findings to broader populations. Additionally, the recruitment strategy (flyers and online announcements) and our inclusion/exclusion criteria may have introduced systematic biases, for example regarding gender or field of study, which should be considered when interpreting the generalisability of the findings. The gender imbalance should be noted, as stress levels and coping mechanisms among college students,^61^ as well as the impact of life stressors on later mental health outcomes,^62^ have been shown to differ by gender. Due to the nature of the sample, cultural factors that may influence the occurrence and meaning of NSSI could not be explored in this context. Including more diverse and cross-cultural samples in future studies would help address this gap. An additional limitation is the cross-sectional, retrospective self-report design which may be subject to recall, consistency or social desirability biases, potentially leading to misreporting of events or behaviours and thereby affecting data reliability. Future longitudinal studies, potentially incorporating third-party data such as clinical documentation, could better examine causal relationships.

### Outlook

This study provides insights into the multifaceted nature of NSSI-related factors in university students. Stable qualitative categories across all three questionnaires highlight the need for mixed-methods approaches to identify clinically significant individual responses beyond the limits of closed-ended questions. Considerable overlaps between qualitative categories and existing closed-ended categories suggest opportunities to refine existing questionnaire items for greater clarity and comprehensiveness. On the other hand, our results highlight the importance of maladaptive coping strategies, trauma and specific NSSI reasons in predicting depression severity. Specifically, coping behaviours such as ‘Auto-aggression’ and ‘Withdrawal/isolation’, as well as NSSI reasons like ‘Termination or reduction of something negative’ and ‘Self-deprecation/-punishment’, were associated with higher depression scores, underscoring the range of factors contributing to depressive outcomes in this population. The qualitatively identified stressor ‘Traumatisation and experiences of violence’ during childhood and adolescence, largely encompassing abuse, should be given special consideration in NSSI-related prevention, diagnostic and treatment, given its impact on both depression severity and NSSI continuation into adulthood. Prevention efforts should focus on early detection of violence or abuse especially within family systems, while diagnostics should incorporate routine screening for traumatising experiences and symptoms in the assessments of individuals presenting with NSSI behaviour. For NSSI patients with a history of traumatic events identified through screening, treatment should address developing skills to manage emotion dysregulation following trauma. Within university health services, our findings support a broader implementation of trauma-informed screening and mental health interventions. Future research should validate our findings in more diverse populations and explore the mechanisms linking trauma to NSSI persistence.

## Data Availability

The data that support the findings of this study are available from the corresponding author, D.S., upon reasonable request.
